# A Unified Approach to Spatial Proximity Query Processing in Dynamic Spatial Networks

**DOI:** 10.3390/s21165258

**Published:** 2021-08-04

**Authors:** Hyung-Ju Cho

**Affiliations:** Department of Software, Kyungpook National University, 2559 Gyeongsang-daero, Sangju-si 37224, Gyeongsangbuk-do, Korea; hyungju@knu.ac.kr

**Keywords:** spatial proximity query, nearest neighbor query, range query, unified batch algorithm, dynamic spatial network

## Abstract

Nearest neighbor (NN) and range (RN) queries are basic query types in spatial databases. In this study, we refer to collections of NN and RN queries as spatial proximity (SP) queries. At peak times, location-based services (LBS) need to quickly process SP queries that arrive simultaneously. Timely processing can be achieved by increasing the number of LBS servers; however, this also increases service costs. Existing solutions evaluate SP queries sequentially; thus, such solutions involve unnecessary distance calculations. This study proposes a unified batch algorithm (UBA) that can effectively process SP queries in dynamic spatial networks. With the proposed UBA, the distance between two points is indicated by the travel time on the shortest path connecting them. The shortest travel time changes frequently depending on traffic conditions. The goal of the proposed UBA is to avoid unnecessary distance calculations for nearby SP queries. Thus, the UBA clusters nearby SP queries and exploits shared distance calculations for query clusters. Extensive evaluations using real-world roadmaps demonstrated the superiority and scalability of UBA compared with state-of-the-art sequential solutions.

## 1. Introduction

This study investigates a unified batch approach to spatial proximity (SP) queries in dynamic spatial networks. In the investigated approach, the distance between two points is the travel time of the shortest path connecting them, and the shortest travel time frequently changes depending on traffic conditions, such as traffic volume and accidents. In this study, SP queries refer to a collection of nearest neighbor (NN) and range (RN) queries, which are basic query types in spatial databases. NN queries retrieve points of interest (POI), such as taxis and restaurants, closest to a query user [1,2], and RN queries retrieve POIs within a query distance [3,4,5]. Typically, location-based services (LBS), such as taxi-booking and ride-sharing services, use real-time spatial data to locate POIs close to the query user [6,7,8,9,10]. When multiple SP queries reach an LBS server simultaneously at peak times, if the SP queries are processed sequentially, it may not be possible to provide prompt responses to the query users. This difficulty can be addressed by increasing the number of LBS servers or by developing state-of-the-art algorithms based on “one-query-at-a-time processing” [3,11,12,13,14,15] to process the SP queries quickly.

SP queries have many potential applications in dynamic spatial networks, such as ride-hailing and car parking facilities. For example, in 2020, the ride-hailing company Uber accomplished an average of 18.7 million trips per day [16], demonstrating the significance of scalable and efficient solutions to promptly match Uber cabs with passengers. Another example is real-time parking management, which helps drivers find a parking space close to their destination.

**Figure 1:**
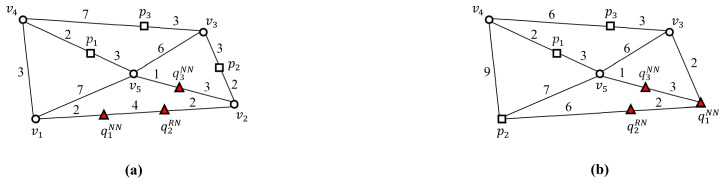
Snapshots of a set of SP queries, *Q* in a dynamic spatial network, where $Q=\{q_{1}^{NN},q_{2}^{RN},q_{3}^{NN}\}$: (**a**) at time $t_{i}$. (**b**) At time $t_{j}$.

Figure 1 shows two snapshots of SP queries in a dynamic spatial network, where a set *Q* of SP query points and a set *P* of data points are expressed as $Q=\{q_{1}^{NN},q_{2}^{RN},q_{3}^{NN}\}$ and $P=\{p_{1},p_{2},p_{3}\}$, respectively. This study assumes that both the query points and data points run freely within the spatial network and that spatial segments often change their weights. In this example, $q_{1}^{NN}$ and $q_{3}^{NN}$ find the data points closest to $q_{1}^{NN}$ and $q_{3}^{NN}$, respectively, and $q_{2}^{RN}$ finds data points within a query distance (e.g., 4 km) to $q_{2}^{RN}$. As shown in Figure 1a, at timestamp $t_{i}$, data point $p_{1}$ is the closest to both $q_{1}^{NN}$ and $q_{3}^{NN}$, and data point $p_{2}$ is within 4 km of $q_{2}^{RN}$. However, as shown in Figure 1b, at timestamp $t_{j}$, data points $p_{3}$ and $p_{1}$ are closest to $q_{1}^{NN}$ and $q_{3}^{NN}$, respectively, and no data point is within 4 km of $q_{2}^{RN}$. A simple solution sequentially retrieves data points that satisfy the condition of each SP query in *Q*. However, this simple solution involves unnecessary network traversal, which can result in prohibitively high computational costs when a large number of SP queries reach the LBS server during peak hours [3,12,13,14,15]. Thus, we propose a unified batch algorithm (UBA) that can process SP queries in dynamic spatial networks effectively and efficiently.

**Figure 2:**
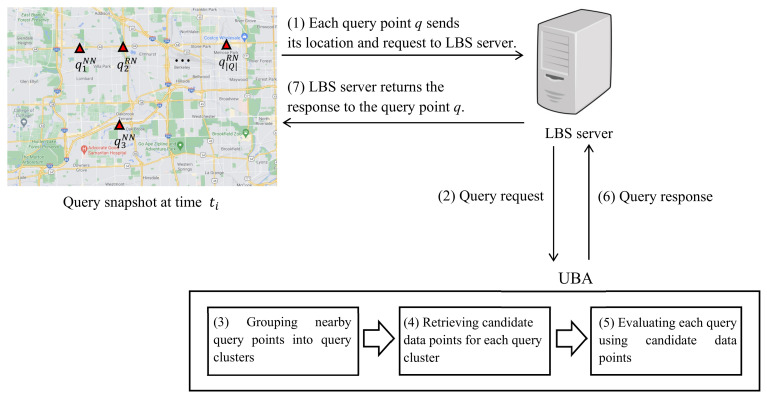
System diagram of UBA between query points and LBS server.

Figure 2 is a system diagram of the proposed UBA between query points and LBS server. Query points send their locations and query requests to the LBS server (step 1). The LBS server collects the requests from query points and forwards them to the UBA (step 2). The UBA first groups nearby query points into query clusters for shared computation (step 3). Then, UBA retrieves candidate data points for each query cluster to avoid unnecessary network traversals (step 4). UBA evaluates each query using the candidate data points for the query cluster (step 5) and returns query results to the LBS server (step 6). Finally, the LBS server provides the result to each query point (step 7).

All nearest neighbor (ANN) queries [17,18] are similar to SP queries. However, ANN queries assume that each query point *q* in *Q* only finds a single data point closest to *q* and, therefore does not consider RN queries. This study considers a highly dynamic situation in which both query and data points run freely within a dynamic spatial network [19,20,21]. The proposed UBA can effectively process SP queries in dynamic spatial networks. For simplicity, this study considers NN queries rather than *k*NN queries, which retrieve *k* data points closest to the query user for a positive integer *k*. However, UBA can easily be extended to process *k*NN queries.

The primary contributions of this study are summarized as follows.

- A unified batch processing algorithm, i.e., UBA, is proposed for the batch processing of SP queries in dynamic spatial networks. The performance of UBA highly depends on the distribution of query points. Thus, UBA clearly outperforms sequential algorithms when query points display a skewed distribution. Conversely, UBA shows similar performance to sequential algorithms when query points display a uniform distribution.
- Clustering of SP queries and their shared computation are presented to avoid unnecessary distance computations. The correctness of UBA is proved using a lemma. Furthermore, a theoretical analysis is presented to establish the advantage of UBA over sequential algorithms, particularly when query points display a skewed distribution.
- An empirical study is conducted under various conditions to demonstrate the superiority and scalability of UBA compared with a sequential algorithm.

The remainder of this paper is organized as follows. Section 2 reviews related studies. Section 3 introduces the necessary preliminaries, including a definition of the notations and symbols used in this study. Section 4 explains how to cluster nearby SP queries into query clusters and presents the proposed UBA for SP queries in dynamic spatial networks. Section 5 presents an empirical study of UBA compared to a conventional algorithm under various conditions. Conclusions and suggestions for future work are presented in Section 6.

## 2. Related Work

Researchers developed algorithms and index structures to evaluate spatial queries, including NN and RN queries for LBSs [6,22,23,24,25]. When calculating the length of the shortest path between two points, spatial queries for dynamic spatial networks suffer from high computational cost because graph traversal is required at runtime. Therefore, numerous studies attempt to reduce the computational cost of the shortest path distance, to avoid unnecessary shortest-path computations [6,22,23,24,25]. Incremental Euclidean restriction (IER) and incremental network expansion (INE) were developed for NN queries [3]. IER assumes that the shortest path between two points is larger than or equal to the Euclidean distance. INE explores the spatial network incrementally from the query point, as in Dijkstra’s algorithm, and investigates the data points in the encountered sequence. Range network expansion (RNE), which is similar to INE, was also developed for RN queries [3]. The route overlay and association directory method, ROAD [12], hierarchically divides the spatial network and pre-calculates the length of the shortest path between the border vertices within each partition. The distance-browsing method, DisBrw [13], exploits the spatially induced linkage cognizance index, and retains the length of the shortest path between each pair of vertices. G-tree [15] hierarchically divides the spatial network and uses an assembly based approach to compute the length of the shortest path between two vertices. V-tree [14] iteratively divides the spatial network into sub-networks and identifies the border vertices of each subnetwork. Then, the V-tree maintains a list of data points closest to each border vertex to quickly evaluate the *k*NN queries. A scalable and in-memory *k*NN query processing method called SIMkNN [26] was developed to quickly evaluate snapshot *k*NN queries over moving objects in a spatial network. The existing methods described in [6,22,23,24,25] are considered to be one-query-at-a-time processing algorithms because they aim to quickly evaluate a spatial query rather than a batch of spatial queries. This study is motivated by the observation that, with simple modifications, NN query processing algorithms can be applied to evaluate RN queries for spatial networks.

Multi-query optimization techniques were originally studied for relational database systems [27]. Their goal is to reduce the computational costs for a collection of queries that concurrently reach the database server by performing shared expressions once, materializing them temporarily, and then recycling them to evaluate other queries. Therefore, the subexpressions are typically evaluated once. These multi-query optimization techniques later expanded to involve query rewriting, query result caches, materialized views, and intermediate query results for relational database systems [28,29,30,31,32,33,34,35,36] and streaming processing systems [37,38,39]. Many applications involving high-load conditions have proven that batch processing algorithms can significantly reduce the query processing time for multiple simultaneous queries [19,30,31,32,33,34,35,36,37,38,39,40,41,42,43]. Furthermore, multi-query optimization techniques have received significant attention in spatial databases. Several batch shortest path algorithms also exist [19,40,41,42,43,44]. Furthermore, multi-query optimization techniques have received significant attention in spatial databases. Several batch shortest path algorithms [19,40,41,42,43,44] have been developed to efficiently evaluate multiple shortest path queries in spatial networks. However, these batch shortest path algorithms cannot be directly used to evaluate SP queries because of their diverse problem definitions. Several cache strategies for query results have been developed to efficiently process batches of *k*NN queries in spatial networks [6]. These strategies exploit the cached results of adjacent recently computed queries to efficiently process a batch of *k*NN queries. However, cache strategies have clear limitations in dynamic spatial networks, as their results may be invalidated by frequent updates to the weight of the spatial segments and by the movement of query points or data points. Finally, Li et al. [45,46,47,48,49] developed a series of algorithms for processing large complex networks, such as social networks. Specifically, they considered the trust management system based on game theory [47], dynamic clustering for electronic commerce systems [45], identifiability for the community detection [49], an optimal estimation of low-rank factors [48], and the identification of overlapping communities [46].

This work differs from existing studies in several respects. First, UBA considers SP queries in dynamic spatial networks. Second, UBA avoids dispensable network traversal by clustering SP queries and performing batch processing. Third, UBA can easily be incorporated into one-query-at-a-time processing algorithms for spatial networks [3,12,13,15].

## 3. Preliminaries

This section defines the terms and notations that are used in this paper.

**Definition** **1** (NN query [1,11,18,22,25])**.** *Given a query point $q^{NN}$ and a set of data points P, an NN query retrieves data point $p_{NN}$ closest to $q^{NN}$ such that dist $\left(q^{NN},p_{NN}\right)\leq dist\left(q^{NN},p\right)$ holds for $\forall p_{NN}\in \Pi \left(q^{NN}\right)$ and $\forall p\in P-\Pi (q^{NN})$*.

**Definition** **2** (RN query [3,4,5])**.** *Given a positive integer r, a query point $q^{RN}$, and a set of data points P, an RN query retrieves data points within query distance r to $q^{RN}$ such that dist $(q^{RN},p_{RN})\leq r$ holds for $\forall p_{RN}\in \Pi \left(q^{RN}\right)$.*

**Definition** **3** (Spatial network [3,9,11,25,26,41,50,51])**.** *A dynamic spatial network can be described as a dynamic weighted graph $G=\langle V,E,W\rangle$, where V, E, and W indicate the vertex set, edge set, and edge distance matrix, respectively. An edge has a nonnegative weight, e.g., travel time, and changes its weight frequently.*

**Figure 3:**
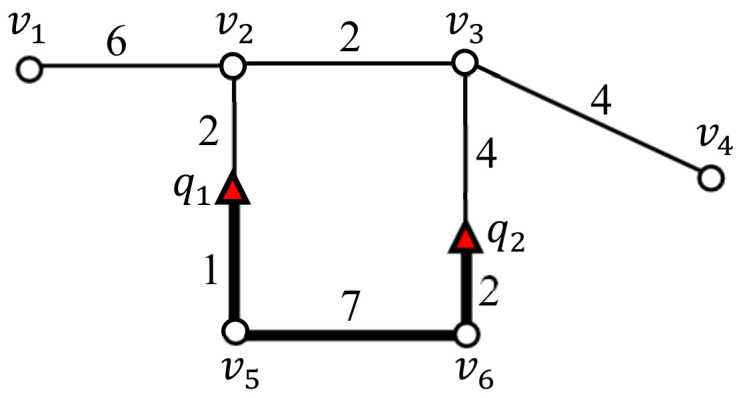
Difference between $dist(q_{1},q_{2})=8$ and $len(\bar{q_{1}v_{5}v_{6}q_{2}})=10$.

**Definition** **4** (Intersection, intermediate, and terminal vertices)**.** *In this study, vertices are categorized via their degree. In this study, vertices are categorized via their degree as follows: (1) if the degree of a vertex is greater than or equal to three, the vertex is an intersection vertex; (2) if the degree is two, the vertex is an intermediate vertex; (3) if the degree is one, the vertex is a terminal vertex. For example, $v_{2}$ and $v_{3}$ in Figure 3 are intersection vertices, $v_{5}$ and $v_{6}$ are intermediate vertices, and $v_{1}$ and $v_{4}$ are terminal vertices.*

**Definition** **5** (Vertex sequence and segment)**.** *A vertex sequence $\bar{v_{l}{v_{l+1}\ldots v}_{m}}$ denotes a segment connecting two vertices $v_{l}$ and $v_{m}$ such that $v_{l}$ and $v_{m}$ are either an intersection vertex or a terminal vertex, and the other vertices in the segment, i.e., $v_{l+1},\ldots {,v}_{m-1}$, are intermediate vertices. The length of a vertex sequence is the total weight of the edges in the vertex sequence. Parts of a vertex sequence are referred to as segments. By definition, a vertex sequence is also a segment. For example, Figure 3 has four vertex sequences $\bar{v_{1}v_{2}}$, $\bar{v_{2}v_{3}}$, $\bar{v_{3}v_{4}}$, and $\bar{v_{2}v_{5}v_{6}v_{3}}$. Examples of query segments in Figure 3 include $\bar{v_{2}v_{5}v_{6}}$, $\bar{v_{5}v_{6}}$ and $\bar{v_{3}v_{6}v_{5}}$.*

Table 1 summarizes the symbols and notations used in this study. Note that the query points are often used interchangeably to refer to SP queries. Figure 3 illustrates the difference between the distance and segment length between $q_{1}$ and $q_{2}$ in a spatial network. Here, the shortest path from $q_{1}$ to $q_{2}$ is $q_{1}\to v_{2}\to v_{3}\to q_{2}$, whose distance $dist(q_{1},q_{2})$ is equal to eight. The segment connecting $q_{1}$ and $q_{2}$ (marked with a bold line) is $\bar{q_{1}v_{5}v_{6}q_{2}}$, and its length $len(\bar{q_{1}v_{5}v_{6}q_{2}})$ is equal to 10.

## 4. Batch Processing of SP Queries in Spatial Networks

### 4.1. Clustering Nearby SP Queries

**Figure 4:**
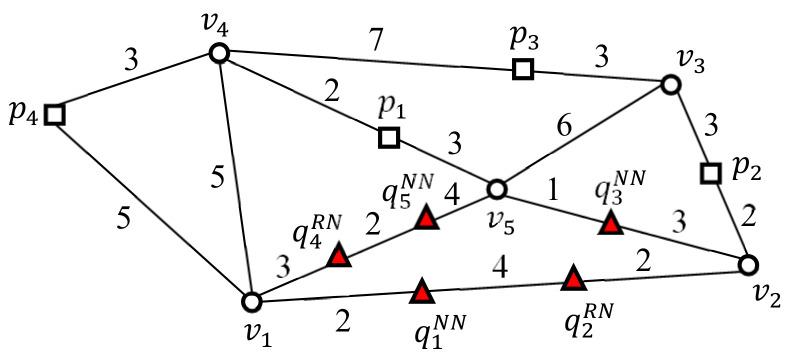
Population comprising five SP queries $q_{1}^{NN},q_{2}^{RN},q_{3}^{NN},q_{4}^{RN},$ and $q_{5}^{NN}$.

Here, we consider five SP queries $q_{1}^{NN},q_{2}^{RN},q_{3}^{NN},q_{4}^{RN}$, and $q_{5}^{NN}$ in a spatial network (Figure 4). Assume that the NN queries $q_{1}^{NN}$, $q_{3}^{NN}$, and $q_{5}^{NN}$ find a data point closest to themselves and that the RN queries $q_{2}^{RN}$ and $q_{4}^{RN}$ find data points within query distance r(=4) to themselves.

**Figure 5:**
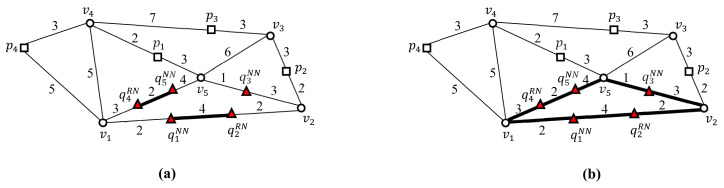
Clustering nearby query points into a query cluster: (**a**) connecting query points in a vertex sequence into a query segment. (**b**) Clustering adjacent query segments into a query cluster using joint vertices.

Figure 5 shows an example of the two-step clustering method, which converts nearby query points into a query cluster. In the first step, query points in a vertex sequence are connected to a query segment (Figure 5a). As a result, three query segments $\bar{q_{1}^{NN}q_{2}^{RN}}$, $q_{3}^{NN}$, and $\bar{q_{4}^{RN}q_{5}^{NN}}$ are generated, where $\bar{q_{1}^{NN}q_{2}^{RN}}$ and $\bar{q_{4}^{RN}q_{5}^{NN}}$ connect two separate sets of query points, i.e., $q_{1}^{NN}$ and $q_{2}^{RN},$ and $q_{4}^{RN}$ and $q_{5}^{NN}$, respectively, in vertex sequences $\bar{v_{1}v_{2}}$ and $\bar{v_{1}v_{5}}$, respectively. In the second step, adjacent query segments are grouped into a query cluster using joint vertices (Figure 5b). The intersection vertex is referred to as a joint vertex when it is adjacent to greater than two query segments. As shown in Figure 5b, query segments $\bar{q_{1}^{NN}q_{2}^{RN}}$ and $\bar{q_{4}^{RN}q_{5}^{NN}}$ are adjacent to an intersection vertex $v_{1}$, which becomes a joint vertex for $\bar{q_{1}^{NN}q_{2}^{RN}}$ and $\bar{q_{4}^{RN}q_{5}^{NN}}$. Similarly, query segments, $\bar{q_{1}^{NN}q_{2}^{RN}}$ and $q_{3}^{NN}$ are adjacent to intersection vertex $v_{2}$, which becomes a joint vertex for $\bar{q_{1}^{NN}q_{2}^{RN}}$ and $q_{3}^{NN}$. Finally, query segments $q_{3}^{NN}$ and $\bar{q_{4}^{RN}q_{5}^{NN}}$ are adjacent to intersection vertex $v_{5}$, which becomes a joint vertex for $q_{3}^{NN}$ and $\bar{q_{4}^{RN}q_{5}^{NN}}$. Therefore, the three query segments, $\bar{q_{1}^{NN}q_{2}^{RN}}$, $q_{3}^{NN}$, and $\bar{q_{4}^{RN}q_{5}^{NN}}$ are connected to a query cluster $\bar{Q_{C}}=\left\{\bar{q_{1}^{NN}q_{2}^{RN}},q_{3}^{NN},\bar{q_{4}^{RN}q_{5}^{NN}}\right\}$. In other words, the five query points $q_{1}^{NN},q_{2}^{RN},q_{3}^{NN},q_{4}^{RN},$ and $q_{5}^{NN}$ are clustered into query cluster $\bar{Q_{C}}$. Note that $\bar{Q_{C}}$ is represented by a set of query segments. Consequently, a set of query points $Q=\{q_{1}^{NN},q_{2}^{RN},q_{3}^{NN},q_{4}^{RN},\;q_{5}^{NN}\}$ is converted into a set of query clusters $\bar{Q}=\left\{\left\{\bar{q_{1}^{NN}q_{2}^{RN}},q_{3}^{NN},\bar{q_{4}^{RN}q_{5}^{NN}}\right\}\right\}$.

Next, we define the border point of query cluster $\bar{Q_{C}}$. Any point at which $\bar{Q_{C}}$ and its non-query cluster $G-\bar{Q_{C}}$ meet is referred to as the border point of $\bar{Q_{C}}$. In this example, $\bar{Q_{C}}$ has three border points, i.e., $v_{1}$, $v_{2}$, and $v_{5}$, where $\bar{Q_{C}}$ and its non-query cluster $G-\bar{Q_{C}}$ meet. Note that sequential solutions should evaluate the five SP queries shown in Figure 4. The two-step clustering method enables UBA to evaluate the three SP queries at border points $v_{1}$, $v_{2}$, and $v_{5}$ rather than at query points $q_{1}^{NN},q_{2}^{RN},q_{3}^{NN},q_{4}^{RN},$ and $q_{5}^{NN}$.

**Figure 6:**
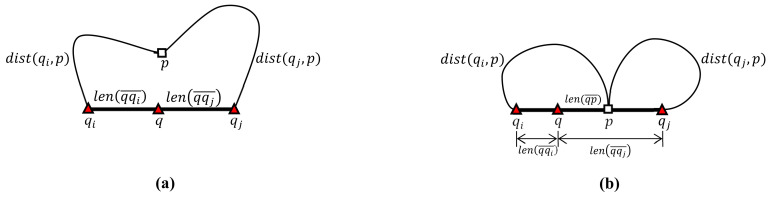
Computation of the distance between query point *q* in query segment $\bar{q_{i}q_{j}}$ and data point *p*: (**a**) $p\notin \bar{q_{i}q_{j}}$. (**b**) $p\in \bar{q_{i}q_{j}}$.

Figure 6 illustrates the computation of the distance between query point *q* in query segment $\bar{q_{i}q_{j}}$ and data point *p* for the following cases: $p\notin \bar{q_{i}q_{j}}$ and $p\in \bar{q_{i}q_{j}}$. As shown in Figure 6a, when data point *p* is outside query segment $\bar{q_{i}q_{j}}$, i.e., $p\notin \bar{q_{i}q_{j}}$, the distance from *q* to *p* is given as $\mathrm{dist}\left(q,p\right)=\min\{\mathrm{len}\left(\bar{qq_{i}}\right)+\mathrm{dist}\left(q_{i},p\right),\mathrm{len}\left(\bar{qq_{j}}\right)+\mathrm{dist}\left(q_{j},p\right)\}$ because the shortest path between *q* and *p* is either $q\to q_{i}\to p$ or $q\to q_{j}\to p$. As shown in Figure 6b, when *p* is inside $\bar{q_{i}q_{j}}$, i.e., $p\in \bar{q_{i}q_{j}}$, the distance is given as $\mathrm{dist}\left(q,p\right)=\min\{\mathrm{len}\left(\bar{qp}\right),\;\mathrm{len}\left(\bar{qq_{i}}\right)+\mathrm{dist}\left(q_{i},p\right),\mathrm{len}\left(\bar{qq_{j}}\right)+\mathrm{dist}\left(q_{j},p\right)\}$ because the shortest path between *q* and *p* is governed by one of the following three cases: q→p, $q\to q_{i}\to p$, or $q\to q_{j}\to p$.

### 4.2. Unified Batch Processing Algorithm for SP Queries

Algorithm 1 provides the key concept of UBA for the unified batch processing of SP queries in a spatial network. Here, the result set Π(Q) is initially set to an empty set (line 1). Then, the nearby query points are first grouped into query clusters (lines 2 and 3), as discussed in Section 4.1. A Cluster search then is executed for each query cluster $\bar{Q_{C}}$ to perform batch processing of the SP queries in $\bar{Q_{C}}$, and its query result is saved to $\Pi (\bar{Q_{C}})$ (line 6). Then, the query cluster result $\Pi (\bar{Q_{C}})$ is appended to Π(Q), where $\Pi \left(\bar{Q_{C}}\right)=\left\{\langle q,\Pi (q)\rangle |q\in \bar{Q_{C}}\right\}$ and $\Pi \left(Q\right)=\left\{\langle q,\Pi (q)\rangle |q\in Q\right\}$ (line 7). When *cluster*_*search* (Algorithm 2) is performed for each query cluster in $\bar{Q}$, UBA terminates by returning the query result Π(Q) (line 8).
**Algorithm 1**UBA(Q,P)**Input**:
*Q*: collection of SP queries, *P*: collection of data points**Output**:
Π(Q): collection of tuples of each SP query *q* in *Q*, and the query result for *q*, i.e., $\Pi \left(Q\right)=\left\{\langle q,\Pi (q)\rangle |q\in Q\right\}$1:Π(Q)←∅ // The result set Π(Q) is initially set to an empty set.2:// Nearby query points are grouped into query clusters, as explained in Section 4.1.3:$\bar{Q}\leftarrow$*cluster*_*points*(Q)  // A set *Q* of query points is changed into a set $\bar{Q}$ of query clusters.4:// *cluster*_*search* function performs a batch processing of SP queries in $\bar{Q_{C}}$, as detailed in Algorithm 2.5:**for** each query cluster $\bar{Q_{C}}\in \bar{Q}$ **do**6: $\Pi (\bar{Q_{C}})\leftarrow$ *cluster*_*search* $\left(\bar{Q_{C}},\;P\right)$       // Note that $\Pi \left(\bar{Q_{C}}\right)=\left\{\langle q,\Pi (q)\rangle |q\in \bar{Q_{C}}\right\}$.7: $\Pi \left(Q\right)\leftarrow \Pi \left(Q\right)\cup \Pi \left(\bar{Q_{C}}\right)$ // The result for query points in a query cluster $\bar{Q_{C}}$, i.e., $\Pi (\bar{Q_{C}})$, is appended to Π(Q).8:**return**Π(Q)      // Π(Q) is returned after the cluster search for all query clusters in $\bar{Q}$ is executed.

Algorithm 2 describes the cluster search algorithm employed to answer SP queries in query cluster $\bar{Q_{C}}$. Here, cluster search performs batch execution for a query cluster to avoid dispensable network traversal. This algorithm runs in two steps. In the first step, the SP queries are evaluated at the border points of $\bar{Q_{C}}$ rather than at the query points in $\bar{Q_{C}}$ (lines 3–6). Note that an SP query is either an NN or RN query; thus, the type of spatial query must be determined, which is evaluated at a border point *b*. If a query cluster $\bar{Q_{C}}$ includes only NN queries, an NN query is evaluated at the border point *b*, i.e., $SPQ\left(b,\bar{Q_{C}}\right)=\Pi \left(b^{NN}\right)$. Similarly, if $\bar{Q_{C}}$ includes only RN queries, the $SPQ(b,\bar{Q_{C}})$ function evaluates an RN query at border point *b*, i.e., $SPQ\left(b,\bar{Q_{C}}\right)=\Pi \left(b^{RN}\right)$. Finally, if $\bar{Q_{C}}$ includes both NN and RN queries, the $SPQ(b,\bar{Q_{C}})$ function evaluates the SP query that finds all the data points satisfying the NN or RN conditions at border point *b*, i.e., $SPQ\left(b,\bar{Q_{C}}\right)=\Pi \left(b^{NN}\right)\cup \Pi \left(b^{RN}\right)$. In the second step, a shared computation is performed for each query segment $\bar{q_{i}q_{j}}$ in $\bar{Q_{C}}$ using the candidate data points obtained at the border points of $\bar{Q_{C}}$ (lines 7–10). Here, each SP query in $\bar{q_{i}q_{j}}$ chooses qualified data points from the candidate data points in $\Pi \left(b_{i}\right)\cup \Pi \left(b_{j}\right)\cup P\left(\bar{b_{i}b_{j}}\right)$, where it is assumed that query segment $\bar{q_{i}q_{j}}$ belongs to segment $\bar{b_{i}b_{j}}$ in $\bar{Q_{C}}$. When the *segment*_*search* (Algorithm 3) is performed for each query segment in $\bar{Q_{C}}$, the *cluster*_*search* algorithm (Algorithm 2) terminates by returning the query result $\Pi (\bar{Q_{C}})$ (line 11).
**Algorithm 2***cluster*_*search*$\left(\bar{Q_{C}},\;P\right)$ **Input:** $\bar{Q_{C}}$: query cluster, *P*: collection of data points **Output:** $\Pi (\bar{Q_{C}})$: collection of tuples of each SP query *q* in $\bar{Q_{C}}$, and the query result for *q*, i.e., $\Pi \left(\bar{Q_{C}}\right)=\left\{\langle q,\Pi (q)\rangle |q\in \bar{Q_{C}}\right\}$1:// Note that $B(\bar{Q_{C}})$ refers to a set of border points in $\bar{Q_{C}}$.2:$\Pi \left(\bar{Q_{C}}\right)\leftarrow \emptyset ,\;\Pi \left(B\left(\bar{Q_{C}}\right)\right)\leftarrow \emptyset$   // Both $\Pi (\bar{Q_{C}})$ and $\Pi (B\left(\bar{Q_{C}}\right))$ are initially set to an empty set.3:// An SP query is evaluated at each border point *b* of $\bar{Q_{C}}$ to retrieve candidate data points for $\bar{Q_{C}}$.4:**for** each border point $b\in B(\bar{Q_{C}})$ **do**5: $\Pi \left(b\right)\leftarrow SPQ\left(b,\bar{Q_{C}}\right)$    // An SP query is evaluated at a border point *b*, and its result is saved to Π(b).6: $\Pi \left(B\left(\bar{Q_{C}}\right)\right)\leftarrow \Pi \left(B\left(\bar{Q_{C}}\right)\right)\cup \Pi \left(b\right)$    // The query result at a border point *b* of $\bar{Q_{C}}$ is appended to $\Pi (B\left(\bar{Q_{C}}\right))$.7:// $\bar{q_{i}q_{j}}$ is assumed to belong to a segment $\bar{b_{i}b_{j}}$ in $\bar{Q_{C}}$.8:**for** each query segment $\bar{q_{i}q_{j}}\in \bar{Q_{C}}$ **do**9: $\Pi \left(\bar{q_{i}q_{j}}\right)\leftarrow segment\_search\left(\bar{q_{i}q_{j}},\;\Pi \left(b_{i}\right)\cup \Pi \left(b_{j}\right)\cup P\left(\bar{b_{i}b_{j}}\right)\right)$    // segment_search is detailed in Algorithm 3.10: $\Pi \left(\bar{Q_{C}}\right)\leftarrow \Pi \left(\bar{Q_{C}}\right)\cup \Pi \left(\bar{q_{i}q_{j}}\right)$       // The result for a query segment $\bar{q_{i}q_{j}}$, i.e., $\Pi (\bar{q_{i}q_{j}})$, is appended to $\Pi (\bar{Q_{C}})$.11:**return return**$\Pi (\bar{Q_{C}})$    // cluster_search ends by returning the batch result $\Pi (\bar{Q_{C}})$ for the SP queries in $\bar{Q_{C}}$.

**Algorithm 3***segment*_*search*$\left(\bar{q_{i}q_{j}},\;\Pi \left(b_{i}\right)\cup \Pi \left(b_{j}\right)\cup P\left(\bar{b_{i}b_{j}}\right)\right)$
 **Input:** $\bar{q_{i}q_{j}}$: query segment in $\bar{Q_{C}}$, $\Pi \left(b_{i}\right)\cup \Pi \left(b_{j}\right)\cup P\left(\bar{b_{i}b_{j}}\right)$: collection of candidate data points of SP queries in $\bar{q_{i}q_{j}}$ **Output:** $\Pi (\bar{q_{i}q_{j}})$: collection of tuples of each query *q* in $\bar{q_{i}q_{j}}$ and the query result for *q*, i.e., $\Pi \left(\bar{q_{i}q_{j}}\right)=\left\{\langle q,\Pi (q)\rangle |q\in \bar{q_{i}q_{j}}\right\}$1:$\Pi (\bar{q_{i}q_{j}})\leftarrow \emptyset$                    // $\Pi (\bar{q_{i}q_{j}})$ is initially set to an empty set.2:**for** each SP query $q\in \bar{q_{i}q_{j}}$ **do**3: Π(q)←∅                     // Π(q) is initially set to an empty set.4: **for** each candidate data point $p\in \Pi \left(b_{i}\right)\cup \Pi \left(b_{j}\right)\cup P\left(\bar{b_{i}b_{j}}\right)$ **do**5:  // Step 1: dist(q,p) is evaluated considering the two cases $p\notin \bar{b_{i}b_{j}}$ and $p\in \bar{b_{i}b_{j}}$, which are shown in Figure 6.6:  **if** *p* is outside $\bar{b_{i}b_{j}}$ **then**7:   $\mathrm{dist}\left(q,p\right)\leftarrow min\{len\left(\bar{qb_{i}}\right)+\mathrm{dist}\left(b_{i},p\right),len\left(\bar{qb_{j}}\right)+\mathrm{dist}\left(b_{j},p\right)\}$  // See Figure 6a.8:  **else**9:   $\mathrm{dist}\left(q,p\right)\leftarrow min\{len\left(\bar{qp}\right),len\left(\bar{qb_{i}}\right)+\mathrm{dist}\left(b_{i},p\right),len\left(\bar{qb_{j}}\right)+\mathrm{dist}\left(b_{j},p\right)\}$    // See Figure 6b.10:  // Step 2: *p* is appended to Π(q) when it satisfies the query condition.11:  **if** $q=q^{NN}$ **and** $dist\left(q,p\right)\leq dist\left(q,p_{NN}\right)$ **then**12:   $\Pi \left(q\right)\leftarrow \Pi \left(q\right)\cup \left\{p\right\}-\left\{p_{NN}\right\}$   // *p* replaces $p_{NN}$ that is the current NN of *q* so far.13:  **else if** $q=q^{RN}$ **and** dist(q,p)≤q.r **then**14:   Π(q)←Π(q)∪{p}       // If dist(q,p)≤q.r, *p* is simply appended to Π(q).15: $\Pi \left(\bar{q_{i}q_{j}}\right)\leftarrow \Pi \left(\bar{q_{i}q_{j}}\right)\cup \Pi \left(q\right)$16:**return**$\Pi (\bar{q_{i}q_{j}})$      // segment_search ends by returning the batch result $\Pi (\bar{q_{i}q_{j}})$ for the SP queries in $\bar{q_{i}q_{j}}$.


Algorithm 3 describes the segment search algorithm employed to answer the SP queries in a query segment $\bar{Q_{C}}$ using the candidate data points in $\Pi \left(b_{i}\right)\cup \Pi \left(b_{j}\right)\cup P\left(\bar{b_{i}b_{j}}\right)$. Here, the batch query result for $\bar{q_{i}q_{j}}$, i.e., $\Pi (\bar{q_{i}q_{j}})$, is initially set to an empty set (line 1). The distance between a query point *q* in $\bar{q_{i}q_{j}}$ and a candidate data point *p*, i.e., *dist*(q,p) is then calculated (lines 5–9), as shown in Figure 6. When *p* is outside $\bar{b_{i}b_{j}}$, i.e., $p\notin \bar{b_{i}b_{j}}$, the distance from *q* to *p* is given as *dist*$\left(q,p\right)=\min\{\mathrm{len}\left(\bar{qb_{i}}\right)+\mathrm{dist}\left(b_{i},p\right),\mathrm{len}\left(\bar{qb_{j}}\right)+\mathrm{dist}\left(b_{j},p\right)\}$. When *p* is inside $\bar{b_{i}b_{j}}$, i.e., $p\in \bar{b_{i}b_{j}}$, the distance from *q* to *p* is given as $\mathrm{dist}\left(q,p\right)=\min\{\mathrm{len}\left(\bar{qp}\right),\mathrm{len}\left(\bar{qb_{i}}\right)+\mathrm{dist}\left(b_{i},p\right),\mathrm{len}\left(\bar{qb_{j}}\right)+\mathrm{dist}\left(b_{j},p\right)\}$. If query point *q* is an NN query and candidate data point *p* is closer to *q* than the current NN $p_{NN}$, then *p* is appended to Π(q) and $p_{NN}$ is removed from Π(q), i.e., $\Pi \left(q\right)\leftarrow \Pi \left(q\right)\cup \left\{p\right\}-\left\{p_{NN}\right\}$ (lines 11–12). Similarly, if query point *q* is an RN query and dist(q,p) is not greater than the query distance q.r, then *p* is simply appended to Π(q), i.e., Π(q)←Π(q)∪{p}, where q.r is the query distance of *q* (lines 13–14). The *Segment*_*search* algorithm (Algorithm 3) ends by returning the batch result $\Pi (\bar{q_{i}q_{j}})$ for $\bar{q_{i}q_{j}}$ (line 16).

Lemma 1 proves the correctness of UBA, which means that each query point *q* in a query cluster $\bar{Q_{c}}$ can retrieve its qualified data points from the candidate data points for $\bar{Q_{c}}$.

 **Lemma** **1.** *Each query point q in a query cluster $\bar{Q_{c}}$ can retrieve its qualified data points from the candidate data points for $\bar{Q_{c}}$.*

**Proof.**  We prove Lemma 1 by contradiction under the assumption that there exists a qualified data point *p* for query point *q* in $\bar{Q_{c}}$ such that *p* is not a candidate data point for $\bar{Q_{c}}$. Clearly, set $\Sigma (\bar{Q_{c}})$ of candidate data points for $\bar{Q_{c}}$ is the union of set $P(\bar{Q_{c}})$ of data points inside $\bar{Q_{c}}$ and the SP query result $SPQ(b,\bar{Q_{c}})$ at each border point of $\bar{Q_{c}}$ as follows: $\Sigma \left(\bar{Q_{c}}\right)=P\left(\bar{Q_{c}}\right)\cup (SPQ\left(b_{l},\bar{Q_{c}}\right)\cup$$SPQ(b_{l+1},\bar{Q_{c}})\cup \ldots$$\cup SPQ(b_{m},\bar{Q_{c}}))$ where it is assumed that $B\left(\bar{Q_{C}}\right)=\left\{b_{l},b_{l+1},\ldots ,b_{m}\right\}$. Clearly, this data point *p* must be outside $\bar{Q_{c}}$. This is because as illustrated in Figure 6b, qualified data point *p* inside $\bar{Q_{c}}$ becomes a candidate data point for $\bar{Q_{c}}$ according to the definition of $\Sigma (\bar{Q_{c}})$. When qualified data point *p* is outside $\bar{Q_{c}}$ as illustrated in Figure 6a, the following two cases should be considered: $\exists p(\left(q^{RN}\in \bar{Q_{c}}\wedge p\in \prod \left(q^{RN}\right)\right)\to$p∉$\Sigma (\bar{Q_{c}}))$ and $\exists p(\left(q^{NN}\in \bar{Q_{c}}\wedge p\in \prod \left(q^{NN}\right)\right)\to$p∉$\Sigma (\bar{Q_{c}}))$. In the first case, i.e., $\exists p((q^{RN}$$\in \bar{Q_{c}}$∧$p\in \prod (q^{RN}))\to$ p∉ $\Sigma (\bar{Q_{c}}))$, qualified data point *p* satisfies the range query $q^{RN}$; however, it is not a candidate data point for $\bar{Q_{c}}$. In the second case, i.e., $\exists p((q^{NN}\in \bar{Q_{c}}\wedge$ $p\in \prod (q^{NN}))\to$ p∉ $\Sigma (\bar{Q_{c}}))$, qualified data point *p* satisfies the NN query $q^{NN}$; however, it is not a candidate data point for $\bar{Q_{c}}$. The shortest path from $q^{RN}$ to *p* should pass through a border point of $\bar{Q_{c}}$. For convenience, assume that the shortest path from $q^{RN}$ to *p* is $q^{RN}\to b_{l}\to p$ where $b_{l}$ is a border point of $\bar{Q_{c}}$. Note that the distance from $q^{RN}$ to *p* is less than or equal to query distance *r*, i.e., $dist(q^{RN},p)\leq r$. Thus, the distance from the border point $b_{l}$ to *p* is also less than or equal to *r*, i.e., $dist(b_{l},p)\leq r$. This leads to a contradiction to the assumption that the qualified data point *p* for $q^{RN}$ is not a candidate data point for $\bar{Q_{c}}$. Next, consider the second case that the qualified data point *p* for $q^{NN}$ is not a candidate data point for $\bar{Q_{c}}$. For convenience, assume that the shortest path from $q^{NN}$ to *p* is $q^{NN}\to b_{l}\to p$ and that a data point $p_{l}$ is the NN of $b_{l}$ rather than *p*. This means that $p_{l}$ is closer to $b_{l}$ than *p*, i.e., $dist\left(b_{l},p_{l}\right)<dist\left(b_{l},p\right)$. Note that the shortest path from $q^{NN}$ to *p* ($p_{l}$) is $q^{NN}\to b_{l}\to p$ ($q^{NN}\to b_{l}\to p_{l}$). Thus, $p_{l}$ should be the NN of $q^{NN}$ rather than *p*. This leads to a contradiction to the assumption that *p* is the NN of $q^{NN}$. Therefore, each query point *q* in a query cluster $\bar{Q_{c}}$ can retrieve its qualified data points from the candidate data points for $\bar{Q_{c}}$. □

Table 2 compares the time complexities of UBA and sequential algorithms, such as INE [3] and RNE [3], for dynamic spatial networks. Note that UBA is independent of the one-query-at-a-time processing algorithms [3,11,12,13,14,15] and can be easily incorporated into these algorithms. For simplicity, INE and RNE are considered to evaluate a single SP query in dynamic spatial networks, and their time complexity is $O(\left|E\right|+\left|V\right|\cdot \log|V|)$. UBA evaluates as many as $M\cdot \left|\bar{Q}\right|$ SP queries, where $\left|\bar{Q}\right|$ is the number of query clusters in $\bar{Q}$ and *M* is the maximum number of border points in $\bar{Q_{C}}$, i.e., $M=max\{|B\left(\bar{Q_{C}}\right)|\;|\;\bar{Q_{C}}\in \bar{Q}\}$. Conversely, sequential algorithms evaluate as many as |Q| SP queries because each query point should be handled individually. Thus, the time complexities of UBA and the sequential algorithms are $O(|\bar{Q}|\cdot (\left|E\right|+\left|V\right|\cdot \log|V|))$ and $O(|Q|\cdot (\left|E\right|+\left|V\right|\cdot \log|V|))$, respectively. The results of the time complexity analysis indicate that UBA is superior to sequential algorithms, particularly when $|\bar{Q}\left|\ll |Q\right|$, i.e., the query points exhibit a highly skewed distribution. In addition, the results demonstrate that UBA shows similar performance to sequential algorithms when $|\bar{Q}\left|≅|Q\right|$, i.e., the query points exhibit a uniform distribution.

### 4.3. Evaluation of Example SP Queries Using UBA

This section describes the process used to evaluate five example SP queries using UBA. As shown in Figure 5, the five SP queries $q_{1}^{NN}$, $q_{2}^{RN}$, $q_{3}^{NN}$, $q_{4}^{RN}$, and $q_{5}^{NN}$ are grouped into a query cluster $\bar{Q_{C}}=\left\{\bar{q_{1}^{NN}q_{2}^{RN}},q_{3}^{NN},\bar{q_{4}^{RN}q_{5}^{NN}}\right\}$, whose border points are $v_{1}$, $v_{2}$, and $v_{5}$. Clearly, a set of query points $Q=\{q_{1}^{NN},q_{2}^{RN},q_{3}^{NN},q_{4}^{RN},\;q_{5}^{NN}\}$ is transformed into a set of query clusters $\bar{Q}=\left\{\left\{\bar{q_{1}^{NN}q_{2}^{RN}},q_{3}^{NN},\bar{q_{4}^{RN}q_{5}^{NN}}\right\}\right\}$. Note that UBA evaluates only three SP queries at the border points of $\bar{Q}$ rather than the five query points $q_{1}^{NN}$, $q_{2}^{RN}$, $q_{3}^{NN}$, $q_{4}^{RN}$, and $q_{5}^{NN}$. Note that $\bar{Q_{C}}$ includes both the NN queries ($q_{1}^{NN}$, $q_{3}^{NN}$, and $q_{5}^{NN}$) and RN queries ($q_{2}^{RN}$ and $q_{4}^{RN}$); thus the results of the SP queries at the border points $v_{1}$, $v_{2}$, and $v_{5}$ should be $\Pi \left(v_{1}\right)=\Pi \left(v_{1}^{NN}\right)\cup \Pi \left(v_{1}^{RN}\right)$, $\Pi \left(v_{2}\right)=\Pi \left(v_{2}^{NN}\right)\cup \Pi \left(v_{2}^{RN}\right)$, and $\Pi \left(v_{5}\right)=\Pi \left(v_{5}^{NN}\right)\cup \Pi \left(v_{5}^{RN}\right)$, respectively. Table 3 shows the results of the SP queries at the three border points $v_{1}$, $v_{2}$, and $v_{5}$.

The *Segment*_*search* algorithm (Algorithm 3) is called for each query segment in $\bar{Q_{C}}$. For convenience, the three query segments $\bar{q_{1}^{NN}q_{2}^{RN}}$, $q_{3}^{NN}$, and $\bar{q_{4}^{RN}q_{5}^{NN}}$ are processed sequentially. First, the *Segment*_*search* function evaluates the SP queries in $\bar{q_{1}^{NN}q_{2}^{RN}}$ with the candidate data points in $\Pi \left(v_{1}^{NN}\right)\cup \Pi \left(v_{1}^{RN}\right)\cup \Pi \left(v_{2}^{NN}\right)\cup \Pi \left(v_{2}^{RN}\right)\cup P\left(\bar{v_{1}v_{2}}\right)=\left\{p_{2},p_{4}\right\}$. This function computes the distance between each pair of query points $q_{1}^{NN}$ and $q_{2}^{RN}$ in $\bar{q_{1}^{NN}q_{2}^{RN}}$, and the candidate data points $p_{2}$ and $p_{4}$. Table 4 summarizes the distances between each pair of query points *q* in query segment $\bar{q_{i}q_{j}}$ and their candidate data points *p*. Here, the SP query $q_{1}^{NN}$ finds the data point closest to $q_{1}^{NN}$ from the candidate data points $p_{2}$ and $p_{4}$. Consequently, $p_{4}$ is the chosen NN of $q_{1}^{NN}$ because $p_{4}$ is closer to $q_{1}^{NN}$ than $p_{2}$ (Table 4). Similarly, the SP query $q_{2}^{RN}$ locates data points within a query distance r=4 to $q_{2}^{RN}$. Accordingly, $p_{2}$ is included in the result of $q_{2}^{RN}$ because $\mathrm{dist}(q_{2}^{RN},p_{2})=4$ and $\mathrm{dist}(q_{2}^{RN},p_{4})=11$ (Table 4). The query result for $\bar{q_{1}^{NN}q_{2}^{RN}}$ is $\Pi \left(\bar{q_{1}^{NN}q_{2}^{RN}}\right)=\Pi \left(q_{1}^{NN}\right)\cup \Pi \left(q_{2}^{RN}\right)=\left\{\langle q_{1}^{NN},\left\{p_{4}\right\}\rangle ,\langle q_{2}^{RN},\left\{p_{2}\right\}\rangle \right\}$.

Next, the *segment*_*search* function evaluates the SP queries in $q_{3}^{NN}$ with the candidate data points in $\Pi \left(v_{2}^{NN}\right)\cup \Pi \left(v_{2}^{RN}\right)\cup \Pi \left(v_{5}^{NN}\right)\cup \Pi \left(v_{5}^{RN}\right)\cup P\left(\bar{v_{2}v_{5}}\right)=\left\{p_{1},p_{2}\right\}$. First, the distance between each pair of query points $q_{3}^{NN}$ and then candidate data points $p_{1}$ and $p_{2}$ is computed. Then, the SP query $q_{3}^{NN}$ locates the data point that is closest to $q_{3}^{NN}$ in $p_{1}$ and $p_{2}$. Consequently, $p_{1}$ is the chosen NN of $q_{3}^{NN}$ because $p_{1}$ is closer to $q_{3}^{NN}$ than $p_{2}$ (Table 4). The query result for $q_{3}^{NN}$ is $\Pi \left(q_{3}^{NN}\right)=\left\{\langle q_{3}^{NN},\left\{p_{1}\right\}\rangle \right\}$.

Finally, the *segment_search* function evaluates the SP queries in $\bar{q_{4}^{RN}q_{5}^{NN}}$ using the candidate data points in $\Pi \left(v_{1}^{NN}\right)\cup \Pi \left(v_{1}^{RN}\right)\cup \Pi \left(v_{5}^{NN}\right)\cup \Pi \left(v_{5}^{RN}\right)\cup P\left(\bar{v_{1}v_{5}}\right)=\left\{p_{1},p_{4}\right\}$. First, the distances between each pair of query points in $\bar{q_{4}^{RN}q_{5}^{NN}}$ and then the candidate data points $p_{1}$ and $p_{4}$ are calculated. The SP query $q_{4}^{RN}$ locates the data points within a query distance r=4 to $q_{4}^{RN}$. No data points belong to the result set of $q_{4}^{RN}$ because $\mathrm{dist}(q_{4}^{RN},p_{1})=9$ and $\mathrm{dist}(q_{4}^{RN},p_{4})=8$ (Table 4). The SP query $q_{5}^{NN}$ identifies the data point that is closest to $q_{5}^{NN}$ in $p_{1}$ and $p_{4}$. Consequently, $p_{1}$ is the chosen NN of $q_{5}^{NN}$ because $p_{1}$ is closer to $q_{5}^{NN}$ than $p_{4}$ (Table 4). The query result for $\bar{q_{4}^{RN}q_{5}^{NN}}$ is $\Pi \left(\bar{q_{4}^{RN}q_{5}^{NN}}\right)=\Pi \left(q_{4}^{RN}\right)\cup \Pi \left(q_{5}^{NN}\right)=\left\{\langle q_{4}^{RN},\emptyset \rangle ,\langle q_{5}^{NN},\left\{p_{1}\right\}\rangle \right\}$. Clearly, the results of the SP queries in *Q* are the union of the results for the query segments in $\bar{Q_{C}}$: Π(Q)= $\Pi (\bar{q_{1}^{NN}q_{2}^{RN}})\cup$ $\Pi (q_{3}^{NN})\cup$ $\Pi (\bar{q_{4}^{RN}q_{5}^{NN}})=$ $\{\langle q_{1}^{NN},\left\{p_{4}\right\}\rangle ,$ $\langle q_{2}^{RN},\left\{p_{2}\right\}\rangle ,$ $\langle q_{3}^{NN},\left\{p_{1}\right\}\rangle ,$ $\langle q_{4}^{RN},\emptyset \rangle ,$ $\langle q_{5}^{NN},\left\{p_{1}\right\}\rangle \}$.

## 5. Performance Study

In this section, the results from an empirical analysis of UBA are presented and compared with those of the conventional method [3]. The experimental settings are described in Section 5.1 and the experimental results are presented in Section 5.2.

### 5.1. Experimental Settings

Three real-world spatial networks [52] (Table 5) were used for the empirical study. These real-world spatial networks have different sizes and are part of the United States road network. For convenience, the extents of the spatial networks were normalized to a unit square ${\left[0,\;1\right]}^{2}$, and the query distance *r* was set to $10^{-2}$. The query points followed a centroid distribution, and the data points followed either a centroid or uniform distribution. Here, centroid-based points were generated to mimic highly skewed distributions of POIs in the real world. First, the centroids $c_{1},c_{2},\ldots ,c_{\left|C\right|}$ were selected randomly based on the extent of the spatial networks, where |C| is to the number of centroids. The points around each centroid followed a normal distribution, with the mean indicating the centroid, and the standard deviation was set to ${\sigma =10}^{-2}$. A total of 1–10 centroids were selected as the query points, and five centroids were selected as the data points. The number of NN queries was the same as that of the RN queries for the SP queries. The experimental parameters are listed in Table 6. In each experiment, a single parameter was varied within the range, and the other parameters were maintained at their default values (shown in bold).

Next, the proposed UBA was compared in terms of query processing time and the number of evaluated SP queries to a sequential algorithm called SEQ, which computes SP queries sequentially. Here, it was assumed that the query and data points moved freely within the dynamic spatial networks. Note that it is impractical to exploit the precomputation techniques presented in the literature [12,13,15] because the precomputed distances might be invalidated frequently when the query and data points run freely within a dynamic spatial network. UBA and SEQ use common subroutines for similar tasks, e.g., the evaluation of SP queries at a single query point; thus, both algorithms were implemented in C++ using the Microsoft Visual Studio 2019 development environment. The experiments were executed on a desktop computer running the Windows 10 operating system with 32 GB RAM and a 3.1 GHz processor (i9-9900). As in many recent studies [11,26,53], the indexing structures for UBA and SEQ remained in main memory to provide prompt responses, which are crucial in online map services. The experiments were repeated 10 times, and the average processing time was measured to determine the SP queries in *Q*. As stated previously, the proposed UBA is orthogonal to one-query-at-a-time processing algorithms [3,11,12,13,14,15] and can be easily incorporated into these algorithms. In this study, INE [3] and RNE [3] were used to evaluate the NN and RN queries, respectively, for the dynamic spatial networks because INE and RNE are based on network expansion similar to Dijkstra’s algorithm, which is well-suited to dynamic spatial networks.

### 5.2. Experimental Results

**Figure 7:**
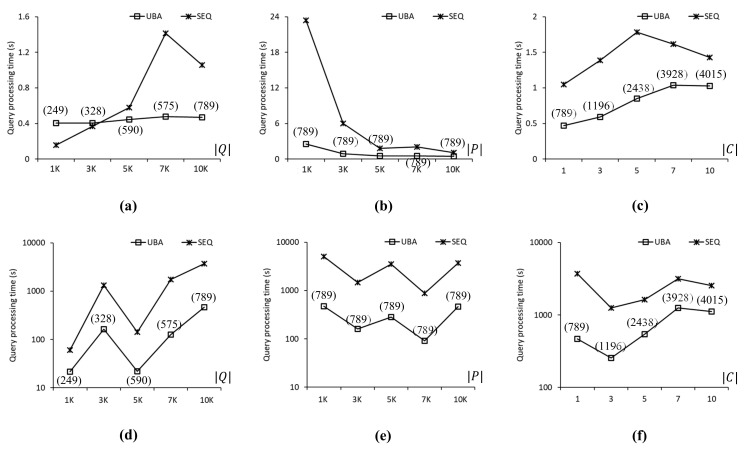
Comparison of query processing times for the CAL roadmap: (**a**) $1\;K\leq \left|Q\right|\leq 10\;K$. (**b**) $1\;K\leq \left|P\right|\leq 10\;K$. (**c**) $1\leq \left|C\right|\leq 10$. (**d**) $1\;K\leq \left|Q\right|\leq 10\;K$. (**e**) $1\;K\leq \left|P\right|\leq 10\;K$. (**f**) $1\leq \left|C\right|\leq 10$.

**Figure 8:**
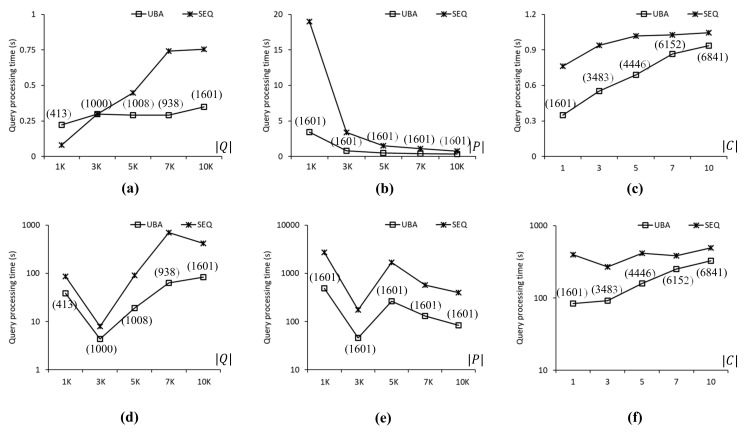
Comparison of query processing times for the FLA roadmap: (**a**) $1\;K\leq \left|Q\right|\leq 10\;K$. (**b**) $1\;K\leq \left|P\right|\leq 10\;K$. (**c**) $1\leq \left|C\right|\leq 10$. (**d**) $1\;K\leq \left|Q\right|\leq 10\;K$. (**e**) $1\;K\leq \left|P\right|\leq 10\;K$. (**f**) $1\leq \left|C\right|\leq 10$.

**Figure 9:**
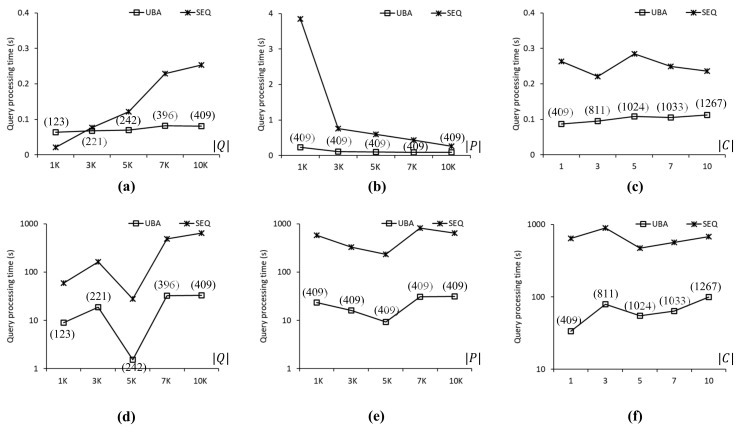
Comparison of query processing times for the COL roadmap: (**a**) $1\;K\leq \left|Q\right|\leq 10\;K$. (**b**) $1\;K\leq \left|P\right|\leq 10\;K$. (**c**) $1\leq \left|C\right|\leq 10$. (**d**) $1\;K\leq \left|Q\right|\leq 10\;K$. (**e**) $1\;K\leq \left|P\right|\leq 10\;K$. (**f**) $1\leq \left|C\right|\leq 10$.

**Figure 10:**
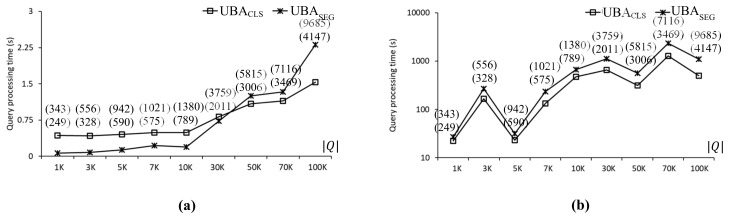
Effect of two-step clustering on the CAL roadmap: (**a**) uniform data points. (**b**) Skewed data points.

Figure 7 compares the query processing times of UBA and SEQ to evaluate the SP queries in the CAL roadmap. In Figure 7, Figure 8 and Figure 9, the three upper-row and three bottom-row charts show the experimental results when the data points followed a uniform distribution and a centroid distribution, respectively. Each chart shows the query processing time and number of evaluated SP queries by varying one parameter at a time (Table 6). The values in parentheses in Figure 7, Figure 8, Figure 9 and Figure 10 indicate the number of SP queries evaluated by the proposed UBA. Note that the numbers of SP queries evaluated by SEQ were omitted because these numbers were exactly equal to $\left|Q\right|$ of the SP queries in *Q*. Figure 7a shows the query processing times of UBA and SEQ when $\left|Q\right|$ of the query points was between 1 K and 10 K, i.e., $1\;K\leq \left|Q\right|\leq 10\;K$. As can be seen, the proposed UBA clearly outperformed SEQ as the number of SP queries in *Q* increased. In terms of query processing times, UBA was up to 2.9 times faster than SEQ for $\left|Q\right|=7\;K$. However, UBA was up to 2.59 times slower than SEQ for $\left|Q\right|=1\;K$. Note that the proposed UBA was not sensitive to $\left|Q\right|$, unlike SEQ, which means that the effectiveness of batch processing in UBA increased as $\left|Q\right|$ increased. When $\left|Q\right|=1\;K$, 3K, 5K, 7K, and 10K, UBA evaluated fewer SP queries than SEQ by 75%, 89%, 88%, 91%, and 92%, respectively. Figure 7b shows the query processing times when |P| of data points was varied between 1 K and 10 K, i.e., $1\;K\leq \left|P\right|\leq 10\;K$. Thus, UBA clearly outperformed SEQ in all cases. The query processing times of UBA were up to 8.9 times lower than those of SEQ when $\left|P\right|=1\;K$. As the $\left|P\right|$ value decreased, the search space for the NN query processing increased. Regardless of the change in $\left|P\right|$, UBA and SEQ evaluated 789 and 10,000 SP queries, respectively. Figure 7c shows the query processing times when |C| of the centroids for the query points was varied between 1 and 10, i.e., $1\leq \left|C\right|\leq 10$. The proposed UBA was up to 2.3 times faster than SEQ for all cases. As |C| increased, the difference in query processing times between UBA and SEQ decreased because increasing |C| led to a reduced density of the query points, which resulted in an increased $|\bar{Q}|$ value. Specifically, when $\left|C\right|=$1, 3, 5, 7, and 10, UBA evaluated 789, 1196, 2438, 3928, and 4015 SP queries, respectively, whereas SEQ evaluated 10 K SP queries for all these cases.

Figure 7d–f show the query processing times of UBA and SEQ when the data points followed a centroid distribution. The query processing times of the proposed UBA were up to 18.95 times lower than those of SEQ for all cases. Unlike the case shown in Figure 7a, the query processing times of UBA and SEQ did not increase with $\left|Q\right|$, as shown in Figure 7d, which means that the query processing time was more sensitive to the distribution of data points than $\left|Q\right|$ when the data points followed a highly skewed distribution. When $\left|Q\right|=1\;K$, 3K, 5K, 7K, and 10K, the query processing times of UBA were 21.7, 162.8, 21.9, 126.8, and 468.7 s, respectively. As shown in Figure 7d–f, UBA was faster than SEQ in all cases. The difference in query processing times between UBA and SEQ for a centroid distribution of data points was up to several orders of magnitude greater than that for a uniform distribution of data points.

Figure 8 compares the query processing times obtained when using UBA and SEQ to evaluate the SP queries in the FLA roadmap. Figure 8a shows the query processing time as a function of |Q|. We found that the proposed UBA was up to 2.2 times faster than SEQ for $\left|Q\right|\geq 3\;K$. However, SEQ was 2.7 times faster than UBA for $\left|Q\right|=1\;K$ because the batch processing of UBA was for a large number rather than a small number of SP queries. Figure 8b shows the query processing time as a function of |P|. UBA was 5.5 and 2.2 times faster than SEQ for $\left|P\right|=1\;K$ and 10K, respectively, even though UBA and SEQ evaluated 1601 and 10,000 SP queries, respectively, for these two cases. This is because the search space for the NN queries when $\left|P\right|=1\;K$ was greater than that when $\left|P\right|=10\;K$. Figure 8c shows the query processing time as a function of |C|, which, for UBA was up to 2.1 times shorter than that of SEQ in all cases. Clearly, the number of query clusters increased with |C|, which adversely affected the performance of the proposed UBA. As shown in Figure 8d–f, UBA was up to 11 times faster than SEQ in all cases. The query processing times of both UBA and SEQ fluctuated, which means that the distribution of highly skewed data points affected the NN query processing time significantly. Specifically, as shown in Figure 8d, the query processing time of UBA for $\left|Q\right|=1\;K$ was 8.9 times longer than that for $\left|Q\right|=3\;K$ despite the difference in the number of SP queries in *Q*.

Figure 9 compares the query processing times obtained using UBA and SEQ with the COL roadmap. As shown in Figure 9a, the proposed UBA was up to 3.1 times faster than SEQ when $5K\leq \left|Q\right|\leq 10\;K$. Here, as $\left|Q\right|$ increased, UBA was superior to SEQ. As shown in Figure 9b, UBA was up to 16.3 times faster than SEQ regardless of the |P| value because UBA and SEQ evaluated 409 and 10,000 SP queries, respectively. Clearly, this difference in the number of evaluated SP queries (i.e., 9591) occurred the proposed UBA can exploit the batch processing of the clustered SP queries; thus, unnecessary distance computations can be avoided. As shown in Figure 9c, UBA clearly outperformed SEQ in all cases of |C|. As |C| increased, the density of the query points decreased, which was ineffective for the batch processing of UBA. As shown in Figure 9d–f, UBA was up to 26.6 times faster than SEQ in all cases. As shown in Figure 9d, the query processing times of UBA and SEQ fluctuated significantly because the highly skewed distributions of data points affected the search space of the NN queries significantly.

Two versions of UBA, i.e., ${\mathrm{UBA}}_{\mathrm{SEG}}$ and ${\mathrm{UBA}}_{\mathrm{CLS}}$, were implemented and evaluated to investigate the effect of the two-step clustering method on the batch processing of UBA and its scalability in terms of $\left|Q\right|$. ${\mathrm{UBA}}_{\mathrm{SEG}}$ transforms nearby query points into query segments, and ${\mathrm{UBA}}_{\mathrm{CLS}}$ transforms nearby query points into query clusters. ${\mathrm{UBA}}_{\mathrm{SEG}}$ and ${\mathrm{UBA}}_{\mathrm{CLS}}$ are illustrated in Figure 5a,b, respectively. Figure 10 compares the query processing times using ${\mathrm{UBA}}_{\mathrm{SEG}}$ and ${\mathrm{UBA}}_{\mathrm{CLS}}$ with the CAL roadmap, where the two values in the parentheses indicate the number of SP queries evaluated by ${\mathrm{UBA}}_{\mathrm{SEG}}$ and ${\mathrm{UBA}}_{\mathrm{CLS}}$, respectively. As can be seen, the number of SP queries evaluated by ${\mathrm{UBA}}_{\mathrm{SEG}}$ was greater than that of ${\mathrm{UBA}}_{\mathrm{CLS}}$. As shown in Figure 10a, when the data points exhibited a uniform distribution, ${\mathrm{UBA}}_{\mathrm{SEG}}$ was up to 6.1 times faster than ${\mathrm{UBA}}_{\mathrm{CLS}}$ for $1\;K\leq \left|Q\right|\leq 10\;K$. However, as $\left|Q\right|$ increased, ${\mathrm{UBA}}_{\mathrm{CLS}}$ was faster than ${\mathrm{UBA}}_{\mathrm{SEG}}$, which means that ${\mathrm{UBA}}_{\mathrm{CLS}}$ scaled better than ${\mathrm{UBA}}_{\mathrm{SEG}}$ with $\left|Q\right|$. Specifically, ${\mathrm{UBA}}_{\mathrm{CLS}}$ was 1.5 times faster than ${\mathrm{UBA}}_{\mathrm{SEG}}$ for $\left|Q\right|=100\;K$. As shown in Figure 10b, when the data points exhibited a centroid distribution, ${\mathrm{UBA}}_{\mathrm{CLS}}$ was up to 2.2 times faster than ${\mathrm{UBA}}_{\mathrm{SEG}}$ in all cases. Therefore, ${\mathrm{UBA}}_{\mathrm{CLS}}$ scaled with $\left|Q\right|$ better than ${\mathrm{UBA}}_{\mathrm{SEG}}$. It is clear that the distribution of data points affected query processing time significantly. Specifically, when the data points exhibited uniform and centroid distributions, the query processing times of ${\mathrm{UBA}}_{\mathrm{CLS}}$ were 1.5 and 497.7 s, respectively, for $\left|Q\right|=100\;K$.

## 6. Conclusions

This paper has proposed the UBA to efficiently process SP queries comprising NN and RN queries in dynamic spatial networks. The goal of the proposed UBA is to avoid dispensable distance computations during batch processing. Accordingly, UBA performs two-step clustering of SP queries and their batch processing to reduce the number of SP queries evaluated for query clusters. The experimental results have confirmed that the proposed UBA outperformed a conventional algorithm based on one-query-at-a-time processing and scaled well with the number of queries. We found that the proposed UBA was up to 26.6 times faster than the compared conventional algorithm. The proposed UBA has several advantages. First, UBA avoids dispensable network traversal by clustering SP queries and performing batch processing. Second, UBA can easily be incorporated into one-query-at-a-time processing algorithms for spatial networks [3,12,13,15]. However, the proposed UBA also exhibits a disadvantage, i.e., its performance is very sensitive to the distribution of query points. Thus, UBA demonstrates similar performance to that of sequential algorithms, particularly when the query points exhibit a uniform distribution. The proposed UBA clearly outperforms sequential algorithms when the query points exhibit a highly skewed distribution. In future, we plan to apply this unified batch solution to extremely large spatial networks for distributed batch processing of sophisticated spatial queries, e.g., spatial join queries [54] and spatial keyword queries [2,50].

## Figures and Tables

**Table 1:** Definitions of symbols.

Symbol	Definition
$q^{NN}$ and $q^{RN}$	NN and RN queries, respectively
*p*	Data point
*r*	Query distance (e.g., 4 km)
*P*	Set of data points
*Q*	Set of query points
$\bar{Q_{C}}$ and $\bar{Q}$	Query cluster and a set of query clusters, respectively
$B(\bar{Q_{C}})$	Set of border points for $\bar{Q_{C}}$
$\Pi (q^{NN})$	Set of data points closest to query point $q^{NN}$
$\Pi (q^{RN})$	Set of data points within query distance *r* from a query point $q^{RN}$
$P(\bar{qp})$	Set of data points in a segment $\bar{qp}$
dist(q,p)	Length of the shortest path connecting points *q* and *p*
$len(\bar{qp})$	Length of the segment $\bar{qp}$ connecting points *q* and *p*
$\bar{v_{l}{v_{l+1}\ldots v}_{m}}$	Vertex sequence where $v_{l}$ and $v_{m}$ are not intermediate vertices, and the other vertices, $v_{l+1},\ldots {,v}_{m-1}$, are intermediate vertices (in short, $\bar{v_{l}v_{m}}$)
$\bar{q_{i}q_{i+1}\ldots q_{j}}$	Query segment connecting nearby query points $q_{i},q_{i+1},\ldots ,q_{j}$ (in short, $\bar{q_{i}q_{j}}$)

**Table 2:** Comparison of time complexities of UBA and sequential algorithms.

	UBA	Sequential Algorithms
Number of SP queries to be evaluated	$M\cdot |\bar{Q}|$	|Q|
Time complexity to evaluate a SP query	$O(\left|E\right|+|V|\cdot \log|V|)$	$O(\left|E\right|+|V|\cdot \log|V|)$
Time complexity to evaluate SP queries in *Q*	$O(|\bar{Q}|\cdot (\left|E\right|+\left|V\right|\cdot \log|V|))$	$O(|Q|\cdot (\left|E\right|+\left|V\right|\cdot \log|V|))$

**Table 3:** Computation of the SP queries at the border points.

Border Point *b*	$\Pi (b^{\mathrm{NN}})$	$\Pi (b^{\mathrm{RN}})$	$\Pi \left(b^{\mathrm{NN}}\right)\cup \Pi \left(b^{\mathrm{RN}}\right)$
$v_{1}$	$\left\{p_{4}\right\}$	∅	$\left\{p_{4}\right\}$
$v_{2}$	$\left\{p_{2}\right\}$	$\left\{p_{2}\right\}$	$\left\{p_{2}\right\}$
$v_{5}$	$\left\{p_{1}\right\}$	$\left\{p_{1}\right\}$	$\left\{p_{1}\right\}$

**Table 4:** Computation of the distances between the queries and the candidate data points.

*q*	*p*	Condition	dist(q,p)	Π(q)
$q_{1}^{NN}$	$p_{2}$	$p_{2}\notin \bar{v_{1}v_{2}}$	$\mathrm{dist}(q_{1}^{NN},p_{2})=8$	$\Pi \left(q_{1}^{NN}\right)=\left\{p_{4}\right\}$
	$p_{4}$	$p_{4}\notin \bar{v_{1}v_{2}}$	$\mathrm{dist}(q_{1}^{NN},p_{4})=7$	
$q_{2}^{RN}$	$p_{2}$	$p_{2}\notin \bar{v_{1}v_{2}}$	$\mathrm{dist}(q_{2}^{RN},p_{2})=4$	$\Pi \left(q_{2}^{RN}\right)=\left\{p_{2}\right\}$
	$p_{4}$	$p_{4}\notin \bar{v_{1}v_{2}}$	$\mathrm{dist}(q_{2}^{RN},p_{4})=11$	
$q_{3}^{NN}$	$p_{1}$	$p_{1}\notin \bar{v_{2}v_{5}}$	$\mathrm{dist}(q_{3}^{NN},p_{1})=4$	$\Pi \left(q_{3}^{NN}\right)=\left\{p_{1}\right\}$
	$p_{2}$	$p_{2}\notin \bar{v_{2}v_{5}}$	$\mathrm{dist}(q_{3}^{NN},p_{2})=5$	
$q_{4}^{RN}$	$p_{1}$	$p_{1}\notin \bar{v_{1}v_{5}}$	$\mathrm{dist}(q_{4}^{RN},p_{1})=9$	$\Pi (q_{4}^{RN})=\emptyset$
	$p_{4}$	$p_{4}\notin \bar{v_{1}v_{5}}$	$\mathrm{dist}(q_{4}^{RN},p_{4})=8$	
$q_{5}^{NN}$	$p_{1}$	$p_{1}\notin \bar{v_{1}v_{5}}$	$\mathrm{dist}(q_{5}^{NN},p_{1})=7$	$\Pi \left(q_{5}^{NN}\right)=\left\{p_{1}\right\}$
	$p_{4}$	$p_{4}\notin \bar{v_{1}v_{5}}$	$\mathrm{dist}(q_{5}^{NN},p_{4})=10$	

**Table 5:** Real-world roadmaps.

Name	Description	Vertices	Edges	Intersection Vertices	Vertex Sequences
CAL	California and Nevada	1,890,815	2,315,222	995,408	1,794,708
FLA	Florida	1,070,376	1,343,951	615,172	1,100,675
COL	Colorado	435,666	521,200	206,069	374,355

**Table 6:** Experimental parameter settings.

Parameter	Range
Number of query points (|Q|)	1, 3, 5, 7, **10** ($\times 10^{3}$)
Number of data points (|P|)	1, 3, 5, 7, **10** ($\times 10^{3}$)
Distribution of query points in *Q*	**(C)entroid**
Distribution of data points in *P*	(U)niform, (C)entroid
Number of centroids for query points in *Q*	**1**, 3, 5, 7, 10
Number of centroids for data points in *P*	**5**
Standard deviation for normal distribution (σ)	$10^{-2}$
Query distance (*r*)	$10^{-2}$
Number of NN queries in *Q*	0.5×|Q|
Roadmap	CAL, FLA, COL

## Data Availability

Not applicable.
